# The clinical value of phase-contrast CMR mitral inflow diastolic parameters: comparison with echocardiography

**DOI:** 10.1186/1532-429X-13-S1-P32

**Published:** 2011-02-02

**Authors:** Emilie Bollache, Alban Redheuil, Carine Defrance, Ludivine Perdrix, Benoît Diebold, Elie Mousseaux, Nadjia Kachenoura

**Affiliations:** 1 1INSERM U678, Paris, France; 2 2Radiology department, European Hospital Georges Pompidou, Paris, France; 3 3Echocardiography department, European Hospital Georges Pompidou, Paris, France

## Purpose

To evaluate the ability of phase-contrast cardiovascular magnetic resonance (PC-CMR) blood flow diastolic parameters to characterize left ventricular (LV) diastolic dysfunction.

## Introduction

Early detection of LV diastolic dysfunction is crucial for the management of patients with heart disease. PC-CMR is increasingly used for this evaluation. However, its usefulness in clinical routine is not established yet because of technical issues such as the lack of automated post-processing tools. We hypothesized that the analysis of velocity and flow-rate curves extracted from an accurate segmentation of the transmitral flow would provide sensitive diastolic parameters.

## Methods

We studied 35 healthy controls (21 women; age: 38±16 years) and 12 consecutive patients (8 women; age: 81±5 years) with a severe aortic stenosis (valve area/body surface=0.47±0.17 cm^2^/m^2^, ejection fraction=66±16%, end-diastolic volume=94±18 ml, end-systolic volume=33±19 ml). All subjects had an echocardiography (GE Vivid 7) and a transmitral flow PC-CMR acquisition (GE 1.5 T) on the same day. For PC-CMR images analysis, we used our custom software for semi-automated segmentation of transmitral flow and automated extraction of diastolic parameters from velocity and flow rate curves. Flow rate curves provided: 1) peak filling rate (Ef_MR_, ml/s) and peak atrial filling rate (Af_MR_, ml/s) combined into Ef_MR_/Af_MR_, 2) peak filling rate to filling volume ratio (Ef_MR_/FVf_MR_, s^-1^), and 3) the deceleration time (DTf_MR_), while maximal velocity curves provided the early and late peak velocities E_MR_ and A_MR_, combined into E_MR_/A_MR_. DT_US_ and E_US_/A_US_ as well as the flow to tissue velocity ratio E_US_/E’_US_ were estimated from Doppler echocardiography.

## Results

A stronger correlation and a slope closer to 1 was found for the comparison between the echocardiographic E_US_/A_US_ and the flow rate-related Ef_MR_/Af_MR_ (r=0.80, Ef_MR_/Af_MR_=0.89·E_US_/A_US_+0.09) than the velocity-related E_MR_/A_MR_ (r=0.72, E_MR_/A_MR_=0.55·E_US_/A_US_+0.49). Results of receiver operating characteristic (ROC) analysis summarized in table 1 indicated the good sensitivity and specificity of the PC-CMR parameters to separate controls from patients.

**Table 1:** Summary of echocardiographic and CMR diastolic parameters for controls and patients and their ability to characterize pathological subjects.  AUC=area under the ROC curve.

	Controls	Patients	Sensitivity	Specificity	AUC
EUS/AUS	1.39 ± 0.60	0.82 ± 0.33	94	67	0.86
EUS/E’US	5.34 ± 1.83	14.3 ± 8.10	83	100	0.96
DTUS(ms)	180 ± 56	271 ± 58	77	92	0.86
EMR/AMR	1.34 ± 56	271 ± 58	77	92	0.86
EfMR/AfMR	1.44 ± 0.58	0.49 ± 0.20	91	92	0.95
DTfMR(ms)	187 ± 36	258 ± 45	86	83	0.88
EfMR/FVFMR (s-1)	4.26 ± 0.94	2.37 ± 0.55	91	100	0.97

## Conclusions

Our automated method provided diastolic parameters in good agreement with Doppler echocardiography especially for our new Ef_MR_/Af_MR_ ratio estimated from flow rate analysis. In addition, our preliminary findings indicated their high sensitivity and specificity to determine patients from controls. The addition of tissue velocities analysis, which is under investigation, to our tool would increase this efficiency.

